# Integrated-Based Curriculum of Pharmaceutical Dosage Forms (ICPDF): What Factors Affect the Learning Outcome Attainment?

**DOI:** 10.3390/ijerph20054272

**Published:** 2023-02-28

**Authors:** Anis Yohana Chaerunisaa, Akhmad Habibi, Muhaimin Muhaimin, Mailizar Mailizar, Tommy Tanu Wijaya, Ahmad Samed Al-Adwan

**Affiliations:** 1 Faculty of Pharmacy, Universitas Padjadjaran, Jatinangor 45363, Indonesia; 2 Fakultas Ilmu Pendidikan dan Keguruan, Universitas Jambi, Jambi 36122, Indonesia; 3 Mathematics Education Department, Universitas Syiah Kuala, Kota Banda Aceh 23111, Indonesia; 4 School of Mathematical Sciences, Beijing Normal University, Beijing 100875, China; 5 Department of Business Technology, Business School, Al-Ahliyya Amman University, Al-Salt 19328, Jordan; 6 Hourani Center for Applied Scientific Research, Al-Ahliyya Amman University, Al-Salt 19328, Jordan

**Keywords:** ICPDF, learning outcome attainment, quality of faculty members, institutional resource, affecting factors

## Abstract

This study aimed to evaluate pharmacy students’ perceptions regarding the correlations among the quality of faculty members, institutional resources, an integrated-based curriculum of pharmaceutical dosage forms (ICPDF), and learning outcome attainment. The current study participants have attended courses (semesters 2 to 6) through the ICPDF in the Department of Pharmaceutics and Pharmaceutical Technology, Faculty of Pharmacy, *Universitas Padjadjaran*, Indonesia. We distributed survey instruments to 212 pharmacy undergraduate students after one year of the curriculum implementation. We asked the students to fill in the instrument in which the indicators consist of a 7-point Likert scale. The data were analyzed using SmartPLS, which included measurement and structural models through PLS-SEM. The findings informed that the quality of faculty members and institutional resources significantly predict ICPDF. Similarly, ICPDF plays a significant role in affecting learning outcome attainment. The quality of faculty members and institutional resources were not related to learning outcome attainment. Significances of differences were informed among students’ years in university regarding learning outcome attainment and ICPDF. However, insignificant differences emerged based on gender. The findings demonstrate the benefits of using the PLS-SEM approach to create a valid and reliable model, assessing the correlations between independent variables with the ICPDF and learning outcome attainment as two dependent variables.

## 1. Introduction

A curriculum evaluation is an integral approach to education that facilitates the foundation for policy decisions of curriculum implementation, especially for feedback on sustainable adjustments and processes [1]. The fundamental concerns regarding curriculum evaluation should refer to the effectiveness and efficiency of the implementation for the education policy and practice, contents, and achievement of the users. Studies have suggested that educational stakeholders keep focusing on evaluating the curriculum implementation [2,3], especially in certain contexts and settings. This study focuses on curriculum implementation in the department of pharmaceutical education.

An integrated curriculum is a curriculum that links different fields of study by cutting across subject matters that focus on a unifying concept. The process might be conducted by combining the subjects studied in an integrated manner, to solve problems according to cases encountered in the field. The integration should aim to make the connections for students so that the learning becomes relevant and meaningful, connected to real life [4]. Integrated curriculums link the theory taught in schools with practical experiences in real-life situations [5]. The idea behind an integrated curriculum is to help students make connections between different subjects and understand how they relate to one another in the real world. For example, an integrated pharmacy lesson might involve teaching students about the medical steps for an urgent situation. An integrated curriculum is important because it helps students develop critical thinking skills, problem-solving skills, and a deeper understanding of the connections between different subjects. When students are able to see how different subjects relate to each other, they are better able to understand and apply what they have learned [4,5]. Additionally, an integrated curriculum can help students see the relevance of what they are learning to the real world, which can increase their motivation to learn.

There have been extensive studies on integrated curriculums, including within medical and pharmaceutical education [4,6,7,8,9]. For example, Al-Eyd et al. [4] demonstrate the utility of curriculum mapping (CM), which depicts the spatial relationships of a curriculum, in the development and administration of an integrated medical curriculum. In their study, a new medical school has created an integrated curriculum based on clinical presentations, that incorporates the active-learning pedagogical practices of numerous educational institutions worldwide. Parallel to the development of the curriculum, a centralized CM process was managed. The findings of their study indicate that CM aided in evaluating content integration, identifying gaps and duplication, linking learning outcomes across all educational levels (i.e., session to course to program), and organizing teaching schedules, instruction methods, and assessment tools [4]. In 2014, Husband et al. [6] informed about how to make an integrated curriculum, in which students are given information in an organized, logical order, but are still asked to make their own connections and learn how to think in an integrated way. A model for an interdisciplinary undergraduate pharmacy curriculum, that is based on facts, is given [6].

An integrated curriculum has been implemented to increase students’ attainment of predetermined learning outcomes [10]. In this study context, we evaluated a 6-month implementation of an integrated-based curriculum of pharmaceutical dosage forms (ICPDF), in one Indonesian public university. The Indonesian pharmaceutical sciences curriculum followed the Indonesian *Standar Nasional Perguruan Tinggi* (National Standard of Higher Education), defined by *Asosiasi Perguruan Tinggi Farmasi Indonesia* (Association of Indonesian Pharmacy Higher Institutions). However, a few studies were conducted to evaluate the implementation of integrated curriculums on the learning outcome attainment within the context of the pharmaceutical program. To fill the gap, this study aims to evaluate the students’ attainment of a predetermined learning outcome after implementing the ICPDF.

## 2. The Study

**Figure 1:**
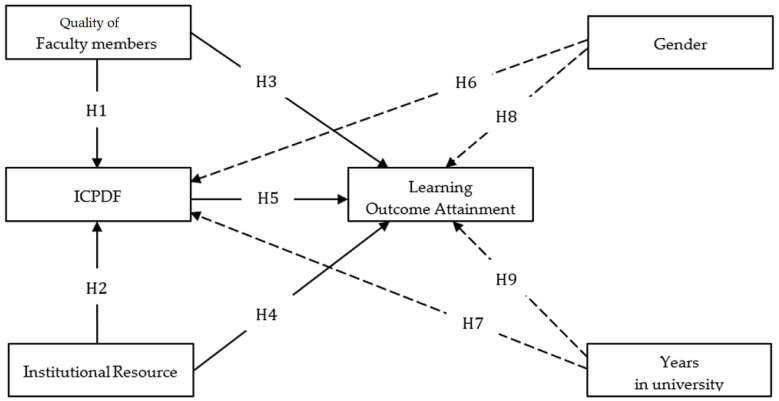
ICPDF evaluation model.

This study involves four constructs: the quality of faculty members, institutional resources, ICPDF, and learning outcome attainment. Demographic information, age, and years at university were also included to understand the differences that emerge on the ICPDF and learning outcome attainment (Figure 1). Previous studies have assessed the quality of the faculty members towards education, training, and research achievement [11]. Quality of faculty members improving teaching quality might foster the level of motivation and the students’ critical thinking [12,13,14,15]. In this study, we investigated whether the quality of faculty members affects the ICPDF and learning outcome attainment. It is important to understand the role of faculty members in bringing the success of curriculum implementation and improving learning outcome attainment.

Similarly, prior studies informed that instructional resources played a significant role in the success of curriculum implementation [3,16]. This study defines an institutional resource as all materials required to support the curriculum implementation. Resources, such as the internet, technology devices, learning spaces and rooms, and laboratory facilities, were examined to understand their influence on the ICPDF and learning outcome attainment. The implementation of the curriculum is a complex process that is achieved through many mechanisms. The learning outcome attainment is described as the wholeness of knowledge, skills, and proficiency that students should have upon the completion of a curriculum. Besides the analysis of factors affecting the ICPDF and learning outcome attainment, demographic information was also included [17] to facilitate data about the research participants, and is needed to determine whether the persons in a particular study are different towards involved factors. Therefore, we include gender and years at university for the additional tests regarding ICPDF and learning outcome attainment, to support the path analysis of the model.

## 3. Learning Outcome Attainment

Learning outcome attainment refers to the knowledge, skills, and abilities that students are expected to possess and exhibit after completing a learning experience or a series of learning experiences. To create a list of learning outcomes, it is crucial to ensure that they are specific and well-defined. This means that the outcomes should clearly and concisely describe the skills that students should be able to demonstrate, produce, and understand because of the program’s curriculum [6,16,17]. It is also important to avoid using ambiguous language and to exclude as many alternative interpretations as possible, to ensure that the outcomes can be accurately measured. In this study, it is the degree to which respondents perceived enhancements of their cognitive knowledge, skills, and abilities after ICPDF.

## 4. The Pharmaceutical Dosage Forms and ICPDF

A Bachelor of Pharmacy graduate is required to have certain competencies that have been determined by the National Standards of Indonesian Higher Education and special competencies determined by the Association of Indonesian Pharmacy Higher Institutions. These competencies have been stated as learning objectives of the curriculum, which must be implemented by all the higher pharmacy institutions in Indonesia. The attainment of the learning outcome that has been determined by the National Standards of Indonesian Higher Education can be obtained by implementing a certain curriculum structure that is expected to accelerate and assist the outcome.

The integrated curriculum structure is one form of curriculum expected to help students understand the learning objectives specified in each course. The integrated curriculum structure also has the advantages of supporting a comprehensive understanding of the knowledge and facilitating graduates to apply and solve problems encountered in the real world, especially in the pharmaceutical field, based on the knowledge in the offered curriculum. The integrated curriculum of this study is part of the curriculum content implemented in the Bachelor of Pharmacy Study program, specifically in pharmaceutical technology, the technology of formulation and manufacture of pharmaceutical dosage forms course. This curriculum content is part of the curriculum provided in the bachelor’s program of pharmacy and other fields of science. Their learning objectives are included as competencies that the graduates must possess. The attainment of the learning objectives from the integrated curriculum is delivered to the students from semester two to semester six, implemented from 2019. Hence its effectiveness in increasing students’ attainment of the targeted learning objectives requires analysis and study through survey and statistical analysis on students as the respondents. We proposed nine hypotheses for the study.

**Hypotheses** **1 (H1).** *The quality of faculty members significantly influences ICPDF.*

**Hypotheses** **2 (H2).** *Institutional resources are significantly related to ICPDF*.

**Hypotheses** **3 (H3).** *The quality of faculty members has a significant role in affecting learning outcome attainment.*

**Hypotheses** **4 (H4).** *Institutional resources significantly influence learning outcome attainment*.

**Hypotheses** **5 (H5).** *ICPDF significantly influences learning outcome attainment*.

**Hypotheses** **6 (H6).** *ICPDF is statistically different based on gender*.

**Hypotheses** **7 (H7).** *Learning outcome attainment is statistically different based on gender*.

**Hypotheses** **8 (H8).** *ICPDF is significantly different based on years at university*.

**Hypotheses** **9 (H9).** *Learning outcome attainment is substantially different based on years at university*.

## 5. Methods

### 5.1. Instrument Establishment and Data Collection

A survey instrument was established to evaluate the ICPDF, adapted from previous studies [3,15]. We discussed the questionnaire with senior faculty members in pharmaceutics and pharmaceutical technology. Four constructs (Table 1), namely the quality of faculty members, institutional resource, ICPDF, and learning outcome attainment, were included to evaluate the perceived opinions of students who have experienced the ICPDF, attending the ICPDF in an academic year, one semester. The indicators were set for self-reporting evaluation on the ICPDF and the learning outcome attainment aiming for the betterment of the curriculum in the future. The initial draft of our survey instrument consisted of forty-three indicators. We implemented a back-translation procedure by translating the scale (English to Indonesian and Indonesian to English). Two translators were invited for the back-translation procedure [18,19].

Initial validation of the survey involves suggestions from users and experts as part of face validity and content validity. We invited four students who previously attended the ICPDF to discuss the instrument as part of the face validity approach. Face validity is conducted when an assessment emerges to do what it claims to do. Therefore, users’ perception regarding the scale established in this study is essential. To support the face validity, we invited two academics in curriculum development and three professors who have experience in teaching pharmaceutical dosage form to discuss the indicators, i.e., content validity [20].

Three indicators were deleted during the face and content validities, while 5 indicators were revised. We deleted 2 indicators because of the unsuitable context and setting; one indicator was deleted because it was indicated as a repetition statement. The five indicators were revised to ease the participants’ understanding of the statements. Therefore, thirty-seven indicators were the final version of the survey instrument (a 7-point Likert scale) for the main data collection.

### 5.2. Participants

The participants of this study were the students who have attended the ICPDF. The current study was an evaluation tool for the ICPDF implementation (one semester/6 months). The inclusion criteria were students of the faculty of pharmacy, *Universitas Padjadjaran,* Indonesia. Printed instruments were shared with the students; the survey was done for three weeks in October 2022. For the sampling, we addressed the use of *G power to determine the sampling numbers [21]. From the *G power assessment, the survey needs 125 or more samples. We collected more responses (n = 212) out of 220 distributed questionnaires given to the participants who attended the course based on ICPDF. The response rate was more than 96% [22]. One hundred and seventy-five respondents were females, while 36 respondents were males. Meanwhile, 149 attended the course in the first year, and 62 were in the second year.

### 5.3. Analysis

Due to the small sample size in the study, PLS-SEM was chosen over covariance-based SEM. PLS-SEM is suitable for analyzing data with limited sample sizes, non-normal distributions, and intricate models with several latent variables and indicators [23,24]. Three steps were conducted to achieve the purposes of the study: the assessment of the measurement, structural model, and demographic difference. The measurement model assessment was conducted through the procedure of the partial least square equation modeling (SEM-PLS), by reporting reflective indicator loadings, internal consistency reliability, convergent validity, and discriminant validity [23,24]. The structural model assessment was also computed using PLS-SEM procedures with the elaboration of path coefficient, t-value, *p*-value, and R^2^. Finally, a *t*-test was conducted to see the differences regarding ICPDF and learning outcome attainment based on age and years at the university of the participants.

## 6. Findings

### 6.1. Measurement Model

For the indicator loadings, the outer loadings should be >0.700 [20]. From the computation, four item loadings were below 0.70. All loading values of less than 0.700 should be subsequently eliminated [25]. The dropped items were ICPDF10 (0.6432), LO8 (0.6940), LO3 (0.6212), and LO1 (0.6878). After the cleaning of the low loadings, 33 items remain for internal consistency reliability (Table 1). The internal consistency reliability focuses on the consistency of computational data across indicators: we examined the construct Cronbach’s alpha and composite reliability (CR) [23]. The values of alpha and CR should exceed 0.700 [23]. The alpha and CR values were reported to be sufficient and exceeded the thresholds (Table 2). The AVE rates are suggested as a measure for determining convergent validity [26]. The PLS-SEM algorithm was used to calculate the outer loadings in the same way. The AVE must be more than 0.500 and must explain 50% or more of the construct elements. All constructs yielded values greater than 0.500 as a result of the computation. As a result, with this measurement, convergent validity should not be a concern (Table 1).

Discriminant validity is defined as the amount to which a construct differs from other constructs in a model. We informed the model’s cross-loading and the heterotrait-monotrait (HTMT) for the discriminant validity. If the loading value on a construct is higher than the values of its cross-loadings on the other constructs, discriminant validity is not an issue [27]. The data disclosed that the outer loadings for every construct were greater than their cross-loadings, establishing the discriminant validity (Table 3). The criteria for HTMT follow the following guidelines: HTMT should be <0.900 [27]. All the HTMT values in Table 4 were less than 0.900. As a result, the relevant construct discriminant validity develops. All valid and reliable items after the measurement model assessment are informed in Appendix A.

### 6.2. Structural Model

**Figure 2:**
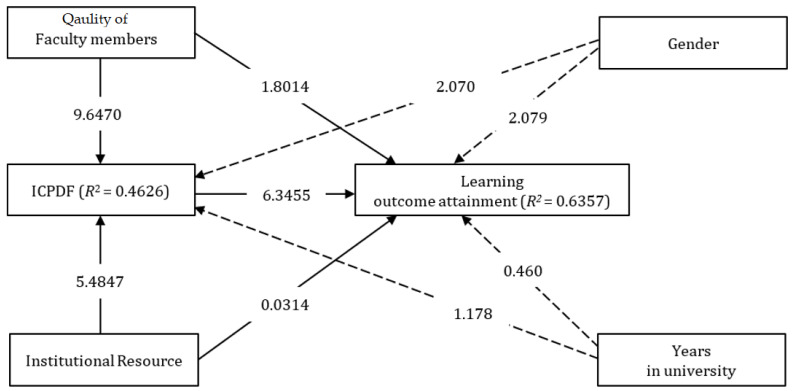
Final framework.

If two or more independent variables in a model are related, it creates redundant information, that is known as multicollinearity. Variance inflation factors should be used to assess multicollinearity in PLS-SEM (VIF). Multicollinearity become a concern when VIF values are greater than 4.0 (Table 4). The predictor sets were evaluated for the VIF values, (1) quality of faculty members and institutional resources as the independent variables of ICPDF; (2) quality of faculty members, institutional resources, and ICPDF as the predictors of learning outcome attainment. Collinearity was not an issue for the model in this study, because all VIF values were less than 4.0. The structural model was used for the reports of the relationships between exogenous and endogenous variables. With a 5% significance threshold, we bootstrapped the data with a sub-sample of 5000 people. We reported that three coefficient correlations were significant. The results of the data computation support H2, H3, and H5. In brief, institutional resources were reported to significantly influence ICPDF (β = 0.3152; *p* < 0.01), supporting H2. Similarly, the quality of faculty members significantly predicted ICPDF (β = 0.5330; *p* < 0.01), which endorses H3. Finally, ICPDF was significant in determining learning outcome attainment (β = 0.5468; *p* < 0.01); thus, this supports H5. Table 4 exhibits the path coefficient (β) and *p*-values (Table 5), while Figure 2 informs the t-values of the data.

Adding the significance test results, effect sizes (f^2^), coefficient determination (R^2^), and predictive relevance (Q^2^) were computed to support the structural model. Effect sizes inform the effect of predictors on dependent variables, symbolized by f^2^. The value of 0.02 was a small effect, 0.15 a medium effect, and 0.35 a large effect [26]. Table 5 informs that the strongest f^2^ was on the relationship between the quality of faculty members and ICPDF (f^2^ = 0.3400), while the smallest f^2^ emerged between institutional resources and learning outcome attainment (f^2^ = 0.0000). The coefficient of determination (*R*^2^) was also computed. *R*^2^ is used to measure predictive accuracy: it is the square correlation between a certain endogenous construct. *R*^2^ values should range from 0 to 1. A higher value shows a higher *R*^2^: 0.75 (substantial), 0.50 (moderate), and 0.25 (weak) [27]. Figure 2 exhibits the result of *R*^2^: ICPDF (*R*^2^ = 0.4626, moderate) and learning outcome (*R^2^* = 0.6357, substantial). In conclusion, the data of this study appeared to be appropriate for the *R*^2^. The study utilized Stone–Geisser’s Q^2^ values to report the results of predictive relevance. Q^2^ is a computational method used in PLS-SEM to evaluate predictive accuracy [27,28]. When the model displays predictive relevance, it can precisely predict the data points of indicators in the model. SmartPLS was used to execute the process of blindfolding. A Q^2^ value greater than 0 indicates that the construct has been accomplished. The Q^2^ values for ICPDF (0.3797) and learning outcome attainment (0.2729) were above 0, indicating that the model’s predictive relevance was good.

### 6.3. Differences Regarding ICPDF and Learning Outcome Based on Age and Years at University

We also investigated whether the demographic information (gender and years at university) is different regarding ICPDF and learning outcome attainment. The number of female students (n = 36) exceeded the number of males (n = 175), which represented the population of the institution. Through a *t*-test, the findings reported that significant differences emerged between the genders, regarding ICPDF (t = 2.070; *p* < 0.05) and learning outcome (t = 2.079; *p* < 0.05). However, years at university were insignificantly different concerning both ICPDF and learning outcome attainment. For the years at university, the sample was not balanced, since the acceptance rate for the 1st year (149) was higher than that of the 2nd year (62). Table 6 presents the details of the *t*-test results.

## 7. Discussion

The main purpose of this study was to analyze how students in the Department of Pharmaceutics and Pharmaceutical Technology, Faculty of Pharmacy, Universitas Padjadjaran, Indonesia, perceived the factors affecting ICPDF and learning outcome attainment after a two-semester integration of the curriculum. Firstly, the proposed framework used in this study was established to evaluate the curriculum based on PLS-SEM and *t*-test analysis. The framework includes a 7-point Likert scale self-administered questionnaire, adapted from prior studies [3,29]. By assessing the face and content validity and the measurement model, 33 valid and reliable items out of the 43 initial items were computed for the structural model and *t*-test. Instrument establishment and development are recommended within an appropriate number of items generated to relate to a study’s setting and context to capture important aspects of the constructs [30]. This study refers to the context and setting in the Indonesian scope and for evaluating an integrated curriculum for Pharmaceutics and Pharmaceutical Technology classes. The valid and reliable items produced by the processes could guide the future evaluation of integrated curriculum offered for researchers with similar interests in various settings and contexts.

Further, this study also reports the significant relationship between the involved variables. Quality of faculty members directly influenced ICPDF, with the most significant association in the structural model. This influence was around twice as strong as the influence of the institutional resources and ICPDF. This implies that the quality of faculty members is important for explaining the curriculum integration in pharmaceutics and pharmaceutical technology after one year of implementation. In other words, experiencing more mentor encouragement in curriculum integration is highly likely to improve alumni perceptions of curriculum integration. A previous study recommended that evaluations of academic programs should be focused not only on curriculums, but also on the faculty members’ activity [31]. Studies have significantly informed that student–faculty-member interactions are crucial for the success of curriculum integration in education [12,13]. Overall, the discussion suggests that the quality of faculty members plays a crucial role in curriculum integration, and their quality and interactions with students can significantly influence the success of the integration process.

However, the present study showed that the quality of faculty members and institutional resources do not play an important role in predicting learning outcome attainment. More studies with various methods should be conducted on why it influences the integration, but not the learning outcome. These kinds of studies can give an insight into considering the recruitment of faculty members in an academic department. Academic departments offering a pharmaceutics and pharmaceutical technology program should encourage faculty members to assess their interactions with learners, qualifications, expertise, and effective learning methods. In academic settings, quality-enhancement practices and institutional resources could be introduced to improve the learning outcome, to help students improve their quality for their future careers. As the quality of faculty members and institutional resources do not predict learning outcome attainment, it is recommended to perform further studies using other approaches to determine why it affects ICPDF, but not the attainment. Academic departments should encourage professors to evaluate their interactions with students, qualifications, knowledge, and effective learning approaches to improve learning outcome attainment.

The curriculum quality in this study, ICPDF, can be defined as a determining factor in improving learning outcome attainment. Students who evaluated the content of the ICPDF as suitable were more often informed about the increase in their learning ability, teamwork, and knowledge. The curriculum was reported to have a significantly positive effect on the students’ performance in related majors. The kinds of instructional approaches at the tertiary level have a significant role in providing students with knowledge and competencies for their professional environment [29,32]. Educational activities engaging students in a structured curriculum might stimulate situations that can improve educational outcomes and prepare students for their future careers. Students could potentially realize that successful learning outcomes are achieved through the combined efforts of the quality of faculty members, institutional resources, and the ICPDF. This implies that relying solely on the quality of faculty members and institutional resources may not yield the same level of success as a properly organized and effectively executed ICPDF.

Besides the instrumentation and the structural model assessment, we also assessed the difference regarding ICPDF and learning outcome attainment to support the findings. Based on the *t*-test, the ICPDF and learning outcome attainment are different considering the gender between males and females. These differences could result in male respondents’ attitudes being more acceptable regarding the ICPDF and learning outcome. However, further studies should be conducted in dealing with this phenomenon. However, insignificant difference emerges from both variables based on years at university. Studies should be carried out regarding demographic information differences for curriculum evaluation, to understand the role of certain curriculum implementation in various contexts and settings.

## 8. Conclusions

In conclusion, the network of associations in the proposed structural model revealed that learning outcome attainment was statistically related to ICPDF. Similarly, ICPDF was significantly predicted by the quality of faculty members and learning outcome attainment. The structural equation model is valuable for curriculum evaluation and obtaining information from students learning the pharmaceutical dosage forms. It allowed faculty members to obtain useful data for internal program design and assess the fit of the educational environment. However, some limitations emerge from the findings of the current study. The study only analyzed data from one academic institution with a focus on pharmaceutical programs, and the variables elaborated might be limited to this area of study. Thus, various contexts and settings of study on the curriculum evaluation are suggested for analysis. The survey design, which was the approach of the study, should be extended: more approaches in data collection, namely interviews, experiments, research and development, are also recommended to perform for future studies. The study has a limitation in terms of sample size, which is relatively small, i.e., less than 500. Thus, to gain a wider perspective, future studies are recommended to involve larger sample sizes. Additionally, due to the cross-sectional nature of the data, it is suggested that alternative and equivalent models be considered by future researchers to investigate other causal effects. The success of an integrated curriculum can be influenced by various factors that can extend the model. Additionally, psychological theories, such as the theory of planned behavior, which involves subjective norms, attitudes, and perceived behavioral intentions, can be integrated into the model to better understand the intention to improve achievement derived from the curriculum.

## Figures and Tables

**Table 1:** Instrumentation.

Construct	Indicators (40)	Definition
Quality of faculty members	F1-F6	Educational and training quality of the faculty members teaching ICPDF
Institutional Resources	IR1-IR6	The infrastructures of the university supporting ICPDF
ICPDF	ICPDF1-16	A course, pharmaceutical dosage forms, implemented an integrated curriculum facilitated by the study program, the Department of Pharmaceutics and Pharmaceutical Technology, Faculty of Pharmacy, *Universitas Padjadjaran*
Learning Outcome Attainment	LO1-LO12	The degree to which respondents perceived enhancements of their cognitive knowledge, skills, and abilities after ICPDF

**Table 2:** Load, alpha, CR, and AVE.

Construct	Item	Load	α	CR	AVE
Quality of faculty members	F1	0.8543	0.9054	0.927	0.6794
	F2	0.8127			
	F3	0.8510			
	F4	0.7880			
	F5	0.8008			
	F6	0.8366			
ICPDF	ICPDF1	0.7865	0.9506	0.9562	0.6097
	ICPDF12	0.7543			
	ICPDF13	0.7054			
	ICPDF14	0.8147			
	ICPDF15	0.8118			
	ICPDF16	0.8310			
	ICPDF2	0.7960			
	ICPDF3	0.7481			
	ICPDF4	0.7867			
	ICPDF5	0.7951			
	ICPDF6	0.7416			
	ICPDF7	0.7437			
	ICPDF8	0.8200			
	ICPDF9	0.7860			
Institutional Resources	IR1	0.7667	0.8872	0.9144	0.6408
	IR2	0.7972			
	IR3	0.8426			
	IR4	0.7465			
	IR5	0.8624			
	IR6	0.7817			
Learning Outcome	LO10	0.7482	0.8966	0.9185	0.6172
	LO11	0.8077			
	LO2	0.7969			
	LO4	0.7386			
	LO5	0.8217			
	LO6	0.7811			
	LO7	0.8012			

**Table 3:** Cross loading.

	Quality of Faculty Members	Institutional Resource	ICPDF	Learning Outcome
F1	**0.8543**	0.6624	0.6763	0.4914
F2	**0.8127**	0.6060	0.6254	0.4491
F3	**0.8510**	0.6707	0.6699	0.4902
F4	**0.7880**	0.5760	0.5944	0.5263
F5	**0.8008**	0.6276	0.5931	0.4167
F6	**0.8366**	0.5714	0.6420	0.5084
ICPDF1	0.6086	0.4785	**0.7865**	0.4982
ICPDF12	0.6614	0.5541	**0.7543**	0.4383
ICPDF13	0.5632	0.6967	**0.7054**	0.4405
ICPDF14	0.6826	0.6535	**0.8147**	0.5267
ICPDF15	0.7000	0.6370	**0.8118**	0.5204
ICPDF16	0.6323	0.5466	**0.8310**	0.6289
ICPDF2	0.6287	0.5317	**0.7960**	0.5259
ICPDF3	0.5515	0.4795	**0.7481**	0.4731
ICPDF4	0.5474	0.5173	**0.7867**	0.5213
ICPDF5	0.5656	0.4727	**0.7951**	0.5635
ICPDF6	0.5169	0.4382	**0.7416**	0.5877
ICPDF7	0.5371	0.5729	**0.7437**	0.5472
ICPDF8	0.5665	0.5505	**0.8200**	0.5653
ICPDF9	0.6165	0.6420	**0.7860**	0.5078
IR1	0.6195	**0.7667**	0.5449	0.4447
IR2	0.6273	**0.7972**	0.5989	0.4289
IR3	0.5690	**0.8426**	0.5795	0.3854
IR4	0.5477	**0.7465**	0.4970	0.3430
IR5	0.6436	**0.8624**	0.6093	0.4569
IR6	0.5920	**0.7817**	0.5956	0.3919
LO10	0.4704	0.4455	0.5754	**0.7482**
LO11	0.5324	0.4396	0.6123	**0.8077**
LO2	0.4621	0.3702	0.5060	**0.7969**
LO4	0.3867	0.3677	0.4142	**0.7386**
LO5	0.4589	0.3968	0.4735	**0.8217**
LO6	0.4156	0.3994	0.4760	**0.7811**
LO7	0.4611	0.3864	0.5958	**0.8012**

Bold, this table shows cross = loading.

**Table 4:** HTMT.

	Quality of Faculty Members	Institutional Resources	ICPDF
Institutional Resources	0.8371		
ICPDF	0.8258	0.7730	
Learning outcome attainment	0.642	0.5699	0.7200

**Table 5:** VIF, β, t-, and *p*-values.

H	Path			VIF	β	*p*-Values	f^2^	Significance
H1	Quality of faculty members	→	Learning outcome attainment	3.0734	0.1651	0.0717	0.0165	No
H2	Institutional resources	→	ICPDF	2.2937	0.3152	*p* < 0.001	0.1189	Yes
H3	Faculty members	→	ICPDF	2.2937	0.5330	*p* < 0.001	0.3400	Yes
H4	Institutional resources	→	Learning outcome attainment	2.5663	−0.0026	0.9750	0.000	No
H5	ICPDF	→	Learning outcome attainment	2.7450	0.5468	*p* < 0.001	0.2026	Yes

**Table 6:** *t*-test.

Construct	Demographic	N	Mean	SD	MD	t-Value	*p*-Value
ICPDF	Female	175	4.8796	0.87639	0.29016	2.070	*p* < 0.05
	Male	36	4.9583	1.19039			
Learning outcome attainment	Female	175	4.6384	0.84374	0.28228	2.079	*p* < 0.05
	Male	36	4.8333	1.16033			
ICPDF	1st	149	4.8078	0.96115	0.29016	0.460	0.646
	2nd	62	5.0979	0.83972			
Learning outcome attainment	1st	149	4.5887	0.89675	0.28228	1.178	0.240
	2nd	62	4.8710	0.90192			

## Data Availability

The datasets generated and/or analyzed during the current study are available from the corresponding author on reasonable request.

## Appendix A

Survey instrument

Gender: Male/Female

Year registered: __________________

**Construct**	**Item**
Quality of faculty members	F1: The faculty members dedicate sufficient time to teach students and support the learning process
	F2: The faculty members have the appropriate qualifications and expertise in occupational pharmacy, suitable for student learning
	F3: The teaching workload allows the faculty members to fully carry out their roles
	F4: The faculty members provide students with a variety of learning methods
	F5: The faculty members have appropriate teaching and research experience for pharmaceutical education
	F6: The faculty members provide innovative methods and thinking for the development of hard skills and soft skills of students in the field of pharmaceutical technology
ICPDF	ICPDF1: Learning objectives (pharmaceutical dosage forms) are clearly communicated in structured material based on the actual process flow in the pharmaceutical industry
	ICPDF12: The meeting sessions for the formulation and pharmaceutical preparation technology subjects (solid, liquid, and semi-solid) were held well
	ICPDF13: A scoring system for each learning objective is sufficient
	ICPDF14: The evaluation system of each course of pharmaceutical preparation technology is well developed
	ICPDF15: Teaching materials regarding the development of pharmaceutical dosage forms contained in the integrated curriculum are adequate
	ICPDF16: When I finished the course of solid, liquid, and semi-solid pharmaceutical formulation and preparation technology delivered in this integrated curriculum, I felt more competent in terms of pharmaceutical technology
	ICPDF2: The curriculum includes materials related to the development of integrated pharmaceutical dosage forms from the pre-formulation stage to the final stage, namely product packaging
	ICPDF3: The curriculum includes material related to the development of pharmaceutical dosage forms in an integrated manner from each related field of study including chemistry, formulation technology and preparation technology from natural ingredients
	ICPDF4: The curriculum can provide a clear overarching picture of the process flow for the development of all types of pharmaceutical dosage forms
	ICPDF5: The integrated curriculum system facilitates student understanding of the description of the development flow of all types of pharmaceutical dosage forms
	ICPDF6: The integrated curriculum system trains students to be problem solvers of problems that arise during the production and evaluation of each type of dosage form
	ICPDF7: The campus environment is very supportive for the application of an integrated curriculum system in the development and production of pharmaceutical preparations
	ICPDF8: Teaching materials regarding the development of pharmaceutical dosage forms contained in the integrated curriculum are very adequate.
	ICPDF9: Laboratory administration systems and study programs support students in implementing a learning system with an integrated curriculum.
Institutional Resource	IR1: Libraries have useful resources, databases and search engines
	IR2: Supporting computer and information technology facilities
	IR3: Laboratory tools support learning and practicum
	IR4: Technical staff are supportive
	IR5: Spaces in the laboratory and faculty buildings support student instructional and practicum activities
	IR6: Chemicals for practicum are provided very adequately
Learning Outcome	LO10: I have the ability to understand and apply the Good Manufacturing Process (GMP) concept, which refers to the CPOB (Good Manufacturing Practices) rules in producing every type of dosage form (solid, liquid, and semi-solid)
	LO11: I have the ability to evaluate the production process flow, quality control, and product packaging of each type of dosage form (solid, liquid, and semi-solid) and provide solutions to problems arising from each process
	LO2: I have the ability to design quality control techniques for raw materials, bulk products, or end products of various types of pharmaceutical dosage forms
	LO4: I have the ability to design a pharmaceutical dosage form (either as a solid, liquid, or semi-solid preparation) of an active medicinal ingredient based on its physicochemical properties
	LO5: I have the ability to develop analysis techniques for the active ingredients of drugs in any kind of pharmaceutical dosage form (solid, liquid, and semi-solid)
	LO6: I have the ability to develop physical and chemical characterization techniques for each type of pharmaceutical dosage form (solid, liquid, and semi-solid)
	LO7: I have the ability to develop a series of process units involved in the production of each type of pharmaceutical dosage form (solid, liquid, and semi-solid) lab-scale and pilot-scale
